# Photoinduced Tunable and Reconfigurable Electronic and Photonic Devices Using a Silk‐Based Diffractive Optics Platform

**DOI:** 10.1002/advs.202000475

**Published:** 2020-06-04

**Authors:** Xiaoqing Cai, Zhitao Zhou, Tiger H. Tao

**Affiliations:** ^1^ State Key Laboratory of Transducer Technology Shanghai Institute of Microsystem and Information Technology Chinese Academy of Sciences Shanghai 200050 China; ^2^ School of Graduate Study University of Chinese Academy of Sciences Beijing 100049 China; ^3^ Center of Materials Science and Optoelectronics Engineering University of Chinese Academy of Sciences Beijing 100049 China; ^4^ School of Physical Science and Technology ShanghaiTech University Shanghai 200031 China; ^5^ Institute of Brain‐Intelligence Technology Zhangjiang Laboratory Shanghai 200031 China; ^6^ Shanghai Research Center for Brain Science and Brain‐Inspired Intelligence Shanghai 200031 China

**Keywords:** circuit reconfiguration, diffractive optical elements, silk, tunable coding metamaterials

## Abstract

A remarkable feature of modern electronic and photonic devices is the ability to maintain their geometric and physical properties in various circumstances for practical applications. However, there is an increasing demand for reconfigurable devices and systems that can be triggered or switched by external stimuli to change geometric, physical, and/or biochemical properties to meet specific requirements such as compact, lightweight, energy‐efficient, and tunable features. Here, a set of phototunable and photoreconfigurable electronic and photonic devices composed of reconfigurable arithmetic circuits and programmable coding metamaterials at terahertz frequencies, empowered by a diffractive optics platform using naturally extracted silk proteins, is reported. These protein‐based diffract optics are precisely manufactured into special microstructures for phase modulation of incident light and can be programmed to degrade at controlled rates. This allows spatial and temporal transformation of the incident light into desired intensity profiles to modulate the electrical properties of multiple photosensitive elements/components within the device simultaneously or discretely. Thus, the optoelectronic functionality of fabricated devices can be tailored to specific applications. Therefore, the approach makes it possible to efficiently fabricate tunable, reconfigurable transient electronic and photonic devices and systems.

## Graphical abstract

A silk diffractive optical element (DOE) based reconfigurable platform is reported. The silk DOE is capable to generate arbitrary 2D patterns and degrade with precisely controlled degradation behaviors, providing multiple degrees of freedom to modulate electrical properties of photosensitive components. Thus, it can be potentially applied in reconfigurable circuits or tunable binary coding metamaterials.

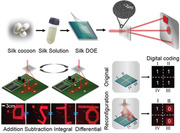

Reconfigurable devices and systems can change shape and multiple desired physical properties upon a variety of external stimuli (e.g., electrical modulation, optical illumination, heat, pressure, and mechanical deformation), providing a new direction for the development of optics,^[^ ^1^, ^2^, ^3^ ^]^ electronics,^[^ ^4^, ^5^, ^6^ ^]^ and photonics.^[^ ^7^, ^8^ ^]^ Reconfigurable devices consisting of components with similar geometric and physical properties are feasible; however, in many cases, it is not practical to fabricate multiple components of different sizes and materials within one manufacturing foundry. Thereby, reconfigurability is often achieved by heterogeneously integrating multiple materials in a composite structure or collectively in a single system, in order to exploit the merit of each material component. For example, such devices and systems can integrate low dimensional materials,^[^ ^9^ ^]^ biologically functional materials,^[^ ^10^ ^]^ plasmonics,^[^ ^11^ ^]^ and metamaterials^[^ ^12^ ^]^ to enable functional transformation from the deformation to changes in a variety of physical and biochemical properties.

However, it still remains challenging to model, functionalize, fabricate, and integrate multiple material components in order to optimize the coupling among mechanical, electrical, optical, thermal, and even chemical properties in such systems to achieve reconfigurability in both spatial and temporal domains.^[^ ^13^, ^14^, ^15^ ^]^ In this regard, it is of paramount importance to select materials that are stimuli‐sensitive, cost‐efficient, easy to fabricate, and functionalize. Silk materials exhibit benefits of relative abundance, low cost, and facile processing into a variety of architectures in addition to their intrinsic biocompatibility and biodegradability, offering opportunities for applications in biologically functional and physically transient devices and systems.^[^ ^16^, ^17^ ^]^

Here, we report a set of phototunable and photoreconfigurable electronic and photonic devices consisting of reconfigurable arithmetic circuits and programmable terahertz coding metamaterials that are enabled by a silk‐based diffractive optics platform. The work builds on the capability to design diffractive optical elements (DOEs) that are optimized for precise light distribution and importantly redistribution on photosensitive elements to rapidly reshape silk with simple, precise patterning techniques.^[^ ^18^ ^]^ In this work, DOEs are used to adjust the phase of incident light to produce the desired intensity profile to modulate the electrical properties (i.e., resistance or conductivity) of multiple photosensitive elements/components within the device simultaneously or discretely, this permits reconfiguration of the output characteristics, the operating frequency, and the transmission/reflection profiles, to be tailored to specific applications.^[^ ^19^ ^]^ Additionally, silk proteins can be physically degenerated in specific environments or degraded in the body at prescribed times and at controlled rates, enabling temporal control of the light distribution impinging on the device to be regulated.^[^ ^20^ ^]^

Upon illuminating diffraction gratings, DOEs generate arbitrary diffraction patterns with different light intensity and distribution that can be used to adjust the performance of the photosensitive components.^[^ ^21^ ^]^ Compared with conventional reconfigurable systems, the silk‐DOE is favored as a photocontrolled, wireless, reconfigurable platform.^[^ ^22^ ^]^ By designing the microstructures of the DOE and adjusting the crystallinity levels of the silk matrix, the spatiotemporal performance of the DOE can be precisely controlled. As shown in **Figure** **1**a, a silk‐DOE with the thickness of ≈46 µm was fabricated using a simple cast‐and‐peel soft lithography process and the crystallinity of silk matrix was controlled via a facile water vapor annealing process (Figures S1 and S2, Supporting Information). After water annealing treatment, the performances of the silk‐DOE can remain stable for several years (Figure S3, Supporting Information). The diffraction patterns of silk‐DOE, with 650 nm illumination were measured at different time points and evaluated by a Red‐Green‐Blue (RGB) color model with 256 color levels (from 0 to 255) in each channel. The *R* values of diffractive patterns (Note: *R*‐values are distinguished from the resistance) measured in red channel were used to assess the characteristics of the silk‐DOE. Note that a 650 nm light source is chosen for proof‐of‐concept and our method is applicable to incident light at other wavelengths depending on applications.

**Figure 1:**
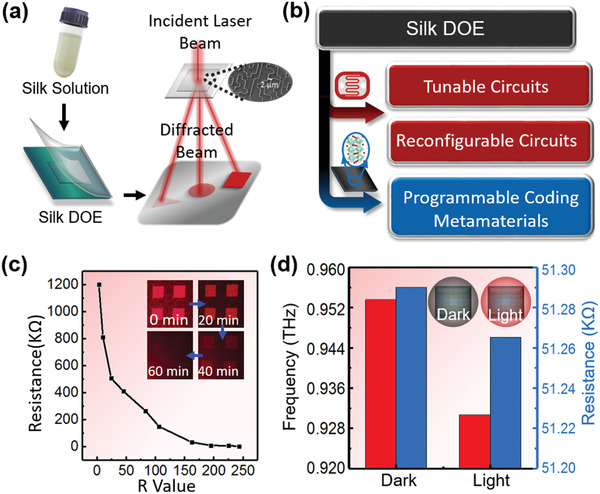
Silk DOE‐based reconfigurable platforms. a) Illustration of fabrication process and working principle of the silk DOE. b) Diffraction patterns with arbitrary 2D light patterns (i.e., shape and intensity) generated from the silk DOE can be used to control the state of photosensitive components (i.e., CdS photoresistors and perovskite films). Adjustable circuits, reconfigurable circuits, and reconfigurable coding metamaterials have been achieved using silk DOE. c) As the silk‐based DOE gradually degrades over time, the integrity of the diffraction pattern is compromised. The resistance of GL5649 photoresistors ranges from a few thousand ohms to megaohms when it is illuminated by diffraction patterns with different *R*‐values. d) The resonance frequency and the resistance of the SRRs measured in dark and light environments.

The design and optimization of the micrograting structures were accomplished by a commercially software LightTrans VirtulLab 5, which can transform the illuminating laser beam into a specified light intensity distribution by diffraction.^[^ ^23^ ^]^ The flexibility of the diffraction pattern and light intensity distribution provides a strategy to effectively and efficiently regulate the properties of the photosensitive semiconductor components. Thus, silk‐DOEs can be used to selectively and dynamically modulate key parameters in a system containing photosensitive components in a spatial and temporal manner. For example, the conductivity or both cadmium sulfide (CdS) and perovskite increases rapidly when illuminated by silk‐DOE; these materials can be used to construct tunable circuits, reconfigurable circuits, and programmable coding metamaterials (Figure 1b).^[^ ^24^, ^25^ ^]^ For instance, the resistance of commercially purchased GL5649 CdS photoresistors in circuits decreased from megaohms (MΩ) to a few kiloohms (kΩ) upon the increase of the *R*‐value for temporal control of the electrical properties of as‐used photoresistors (Figure 1c). A series of split ring resonators (SRRs) coated with the perovskite and a silk‐DOE that provides reconfigurable coding metamaterials were measured in dark and light environments, respectively. The resonance frequencies and the resistances of the SRRs were measured in dark and light environments, respectively, to show the effect of silk‐DOE on photosensitive components directly. As shown in Figure 1d, the resonance frequency exhibits a small redshift and the resistance is slightly reduced.

To demonstrate the circuit tunability provided by silk‐DOE, it is necessary to verify the capability of the silk‐DOE to hold and regulate the outputs dynamically. As shown in **Figure** **2**a, an adder circuit was composed of an OP27 series precision operational amplifier, five commercially purchased fixed resistors and one photoresistor. As the surrounding ambient humidity was increased from 40% to 55%, R4 exhibited a decrease over hundreds of kΩs, inducing an obvious output deviation (Figure S4, Supporting Information). In order to compensate for the decrease of R4 and thereby maintain a stable output, the resistance of R5 should be increased synchronously with the increase of environmental humidity. Therefore, R5 was illuminated in real‐time by a silk‐DOE (with a 3 h water annealing process in this case to control the crystallinity thus the degradation rate) that had been degraded for different times by immersing into a 0.3 mg mL^−1^ proteinase K solution to acquire proper *R*‐values (Figure S5, Supporting Information). Thus, the decrease of R4 was offset by R5 resulting in relatively stable output voltages for different relative humidities, as shown in Figure 2b. In addition to maintaining the function of the adder circuit, the *R*‐values corresponding to a diffraction pattern in response to variable relative humidities can be measured and correlated to relative humidity (Figure 2c).

**Figure 2:**
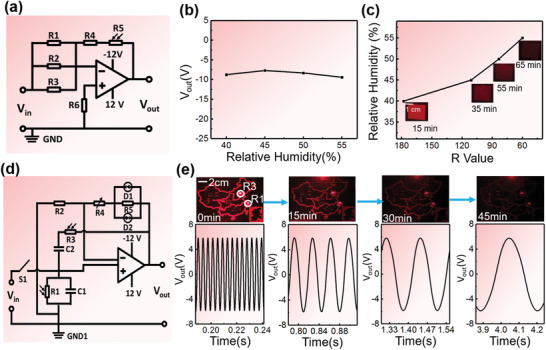
Silk DOE used for tuning circuit output characteristics. a) Schematic diagram of the adder circuit. R1, R2, R3, and R6 are fixed resistors with resistance values of 300, 300, 300, and 75 kΩ, respectively. R4 and R5 are humidity‐sensitive and photosensitive resistors, respectively. The resistance of R5 was be regulated by a silk DOE. With *V*_in_ at 3 V in the adder circuit, the calculated *V*_out_ is 9 V. b) As the relative humidity increased from 40% to 55%, the *V*_out_ of the adder circuit almost remains steady through the compensation of the photoresistor, R5. c) The relationship between *R*‐value of the diffraction pattern and the ambient humidity as the silk DOE degraded for different time (15, 35, 55, and 65 min). The relative humidity can be determined from the *R*‐value through the calibration curve. d) Schematic diagram of the Resistor‐Capacitor (RC) oscillator circuit. e) The resonance frequency of the oscillator circuit was modulated by regulating the resistance value of the photoresistors in the selection network, realized by adjusting the degradation time of the silk DOE. For degradation times of 0, 15, 30, and 45 min, the resonant frequencies were 253.55, 28.4, 7.85, and 3.18 Hz, respectively.

Moreover, silk‐DOE can be used to alter key circuit parameters^[^ ^26^ ^]^ by regulating the resistance of several CdS photoresistors simultaneously or sequentially. A typical oscillation circuit has a fixed resonance frequency due to the frequency selection network, but the demand for circuits with an adjustable frequency is increasing. In order to exploit a circuit with adjustable resonance frequencies, we have designed a tunable oscillation circuit which is composed of a UA741 precision operational amplifier, three capacitors (C1, C2 = 0.1 µF) and nine resistors with resistance values of 2.0 kΩ (R1) and 2.2 kΩ (R2). R3 uses an adjustable resistor to meet the oscillation‐starting conditions for the circuits, and R4 and R5 are GL5649 photoresistors, respectively (Figure 2d). In this circuit, the feedback link is composed of R1, R2, and R3, while R4, C2, R5, and C3 form both the positive feedback and frequency selection network. The frequency of this circuit satisfies the function $f=\frac{1}{2\pi \sqrt{R4\cdot R5\cdot C2\cdot C3}}\;$. A diffraction pattern of “a map of China” which overlaps with a printed circuit board (PCB) was devised to modulate the R4 and R5 controlled by the light spots illuminated on “Beijing” and “Shanghai,” respectively (Figure S6, Supporting Information), indicating that frequency selection network can be regulated temporally by controlling the degradation process of silk‐DOE. A silk‐DOE, annealed in water vapor for 3 h, was progressively degraded in proteinase in a 0.3 mg mL^−1^ K solution to create a series of decreased *R*‐values; these decreased resistances evoke increasing resistance of R4 and R5 to generate the corresponding diminished resonance frequency. In our experiment, when the degradation time points were set to 0, 15, 30, and 45 min, the resonance frequencies of adjustable oscillation circuit were regulated to 253.55, 28.40, 7.85, and 3.18 Hz, respectively (Figure 2c).

Based on the ability of silk‐DOEs to both maintain the output of circuits and to alter their characteristic parameters, we developed a novel method for fabricating photoinduced reconfigurable circuits.^[^ ^27^ ^]^ This involved using a silk‐DOE to spatially modify circuits (**Figure** **3**a). Circuits can be reconfigured for different functions by changing the diffraction gratings of a silk‐DOE to generate different circuit layouts (Figure S7, Supporting Information). All the photoresistors can be switched from “off” to “on” states by light; in the “dark” (i.e., illuminated only by stray light) their resistance values remain in the MΩ range which defines the “off” state but when they are illuminated by diffracted light, their resistance values decrease to several kΩs, triggering the “on” state. Thus, when the silk‐DOE is illuminated with a preset intensity at 650 nm, the photoresistors located in areas where the diffracted beam impinges on the circuit are turned “on,” resulting in circuit reconfiguration. To implement and test this strategy, a reconfigurable circuit—in which all resistors were GL5649 photoresistors—was designed and fabricated on a PCB. Four diffraction patterns were designed to overlap with four distinct combinations of photoresistors (some of which were common) on the same PCB (Figure 3b and Figure S8, Supporting Information). In this manner, four different set circuits of adder, subtractor, integrator, and differentiator could be implemented and tested on a single system (Figure 3c and Figure S9, Supporting Information).

**Figure 3:**
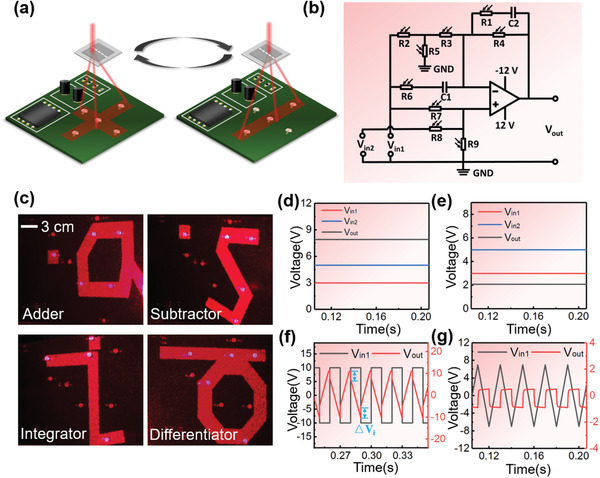
Silk DOE‐programmed circuit reconfiguration. a) Schematic illustration of silk DOE induced reconfigurable circuits. b) Schematic diagram of a circuit which can be reconfigured into adder, subtractor, integrator, and differentiator circuits. In the reconfigurable circuit, all resistors are GL5649 photoresistors. c) Adder, subtractor, integrator, and differentiator circuits are constructed using four diffraction patterns (i.e., in the shapes of “a, s, i, d,” respectively) to control the relevant photoresistors. d–g) The output characteristics of the adder, subtractor, integrator, and differentiator circuits. d) Adder circuit: *V*_in1_ and *V*_in2_ are set to 3 and 5 V, respectively, and the *V*_out_ (*V*_out_ = *V*_in1_ + *V*_in2_) is ≈7.89 V. e) Subtractor circuit: *V*_in1_ and *V*_in2_ are set to 3 and 5 V, respectively, and the *V*_out_ (*V*_out_ = *V*_in2_ − *V*_in1_) is ≈2.081 V. f) Integrator circuit: The input *V*_in1_ signal is a square wave with a duty cycle of 50% (black line) and a repetition frequency of 50 Hz. The *V*_out_ is an approximate triangular wave (red line) with a voltage jump, Δ*V*_i_. g) Differentiator circuit: *V*_in1_ is a triangular wave (black line) with a duty cycle of 50% and a frequency of 50 Hz. The *V*_out_ output signal is an approximate square wave.

When R3, R4, R5, R7, and R8 are illuminated, they comprise of part of the adder circuit, in which *V*_in1_ (red line) and *V*_in2_ (blue line) are set as 5 and 3 V, respectively, and the *V*_out_ (*V*_out_ = *V*_in1_ + *V*_in2_) (black line) is ≈7.89 V (Figure 3d). Resistors R2, R3, R4, R8, and R9 comprise the subtractor circuit; in this case, *V*_in1_ (red line) and *V*_in2_ (blue line) are set as 3 and 5 V, respectively, and the *V*_out_ (*V*_out_ = *V*_in2_ – *V*_in1_) (black line) is ≈2.08 V (Figure 3e). The *V*_out_ (black line) of the integrator circuit is shown in Figure 3f, in which the ideal output should be a triangular wave when *V*_in1_ (red line) is square wave. A voltage jump written as Δ*V*_i_ (Δ*V*_i_ = *i*·R1, the positive direction of *i* is the current direction flowing in from *V*_in1_) occurs at the peak of the triangular wave of *V*_out_ that satisfies Equation (1), mainly because R1 does not have the same properties of actual wire. In addition, *V*_out_ jumps occur at the valley of triangular wave when both *V*_in1_ and Δ*V*_i_ are negative. When *V*_in1_ is positive with positive Δ*V*_i_, a downward movement occurs at the valley of triangular wave of *V*_out_. When the circuit is reconfigured to the differential mode with a triangular input wave (*V*_in1_), the waveform (black line) is an approximate square wave (Figure 3g). This output waveform was analyzed using Equation (2), in which Δ*V*_d_ mainly results from a voltage drop due to R6. When *V*_in_ changes at a constant rate, capacitor C1 will undergo a charge/discharge process during which the variation ratio of current will decrease gradually. As a result, the variation ratio |Δ*V*_d_| declines, leading to an increase of |*V*_out_|. To reduce the error of output signal, a significant way is to improve the signal‐ratio‐noise such as decreasing the critical feature size or increasing the phase level (Figures S10 and S11, Supporting Information).

(1) $$\begin{array}{c} -\frac{1}{\left(R2+R3\right)\cdot C2}\int V_{\mathrm{in}1}\cdot dt=\;V_{\mathrm{out}}+\Delta V_{i} \end{array}$$

(2) $$\begin{array}{c} -R4\cdot C1\cdot \frac{d\left(V_{\mathrm{in}\;1}-\Delta V_{d}\right)}{dt}=V_{\mathrm{out}} \end{array}$$

To further explore the applications of silk‐DOEs in digitally programmable coding metamaterials, we combined photosensitive materials with the sub‐wavelength metamaterial elements of SRRs.^[^ ^28^, ^29^ ^]^ THz spectroscopy is used to detect small changes in the conductivity of the coating on the SRRs for photoinduced programmable and reconfigurable binary coding metamaterials.^[^ ^30^ ^]^ Incorporation of such photosensitive materials into the resonator system can alter the capacitance (*C*) and/or inductance (*L*) of the system, thus modifying its resonance frequency, *ω* = 1/*LC*.

As shown in **Figure** **4**a, a CH_3_NH_3_PbI_3_ coating, which is a commonly used photosensitive material (Figure 4b and Figure S12, Supporting Information), was deposited on gold SRRs using a previously reported one‐step method.^[^ ^31^, ^32^ ^]^ For these SRRs with a lateral dimension and periodicity of 43 and 86 µm, respectively (Figure 4c), the *LC* resonance will be ≈1.0 THz (Figure S13, Supporting Information). The microstencils with desired metamaterials structures were fabricated on a commercially purchased 4 in. high‐resistivity silicon wafer using the metal lift‐off technique (Figure S14, Supporting Information). As shown in Figure 4d, simulations show that the *LC* resonance exhibits a redshift^[^ ^33^, ^34^ ^]^ due to the increase of capacitance and conductivity in CH_3_NH_3_PbI_3_ coating going from the illuminated to dark state. To demonstrate and construct binary coding metamaterials, we established a specific model composed of binary digital elements of “0” or “1” by dividing the CH_3_NH_3_PbI_3_‐coated SRRs into four parts (I, II, III, and IV) (Figure 4e). The “0” elements are defined as the parts that are regulated by diffractive patterns while the “1” elements are the parts whose parameters are not influenced by illumination. In these binary coding metamaterials, the coding sequence can be reconfigured by switching with one silk‐DOE or between different silk‐DOEs. The transmission spectra of regions II and III were experimentally measured; as shown in Figure 4f, these regions exhibit a redshift of about 200 GHz in the light environment, upon selective illumination by the silk‐DOE, while no significant wavelength shift in the transmission spectra of nonilluminated regions was observed. The second derivative of measured transmission spectra at 1.0 THz was set as the coding criteria in which the coding parts were defined as “0” (over 700) and “1” (below 700) and the numerical coding was successfully reconfigured from “1111” to “1010” by adjusting the silk‐DOE (Figure 4g).

**Figure 4:**
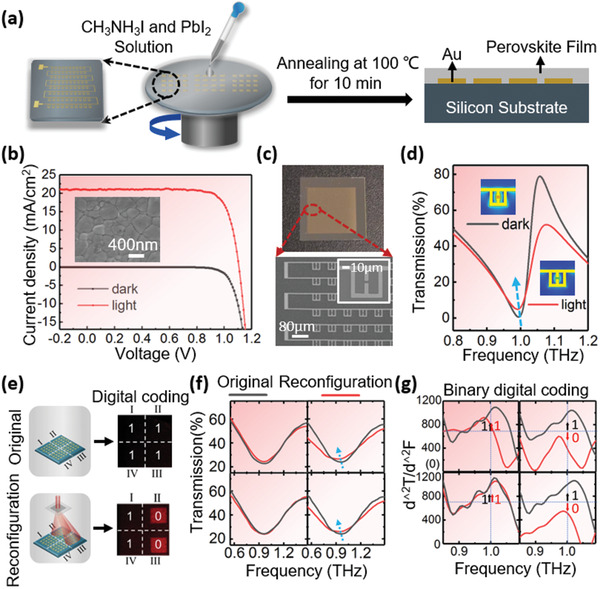
Digitally reconfigurable binary coded metamaterial realized by silk DOE. a) Fabrication process of CH_3_NH_3_PbI_3_‐coated metamaterials used as reconfigurable binary digital elements. b) Measured current density–voltage curves of CH_3_NH_3_PbI_3_‐based photovoltaic device under dark and illuminated (at simulated AM 1.5G solar irradiation with light intensity of 100 mW cm^−2^) conditions, respectively. c) Photograph and scanning electron microscope (SEM) image of the metamaterials without CH_3_NH_3_PbI_3_ coatings. d) Calculated transmission spectra of the CH_3_NH_3_PbI_3_‐coated metamaterials in dark and light conditions, respectively. (Inset) Simulated electric field enhancement distribution at the resonance frequency. e) Schematic of reconfiguration of the binary coded digital metamaterial. The coding sequence is defined by the diffraction pattern produced by the silk DOE. f) Measured transmission spectra of the “binary digital elements” before and after silk DOE‐induced reconfiguration (panels correspond to Roman numeral labeled panels in (e)). g) Binary coding the metamaterials using the second derivative of the measured transmission spectra. Second derivative values over 700 and below 700 at 1 THz are considered as “0” and “1” states, respectively (panels correspond to Roman numeral labeled panels in (e)).

In summary, we have designed and fabricated a set of silk‐based DOEs that serve as light modulators to reconfigure photosensitive electronic and photonic devices by precisely distributing and redistributing light. As a proof‐of‐concept, we have demonstrated a set of reconfigurable arithmetic circuits with dynamically tunable performances including output characteristics, operating frequency as well as the operation modes (i.e., adder, subtractor, integrator, and differentiator) within one single circuit layout which can be facilely switched between different modes using the silk DOEs. Furthermore, by adjusting the degradation process and thus the diffractive patterns of silk DOEs, we can precisely control the resistance of the photoresistors to compensate for the output drift of circuit elements caused by environmental variations (e.g., humidity). Moreover, we have demonstrated the photoregulation of binary coding metamaterials (switched between “0” and “1” by photomodulation of transmission spectra of metamaterials at terahertz frequencies) with the aid of a photosensitive perovskite layer. Thus, an innovative strategy has been developed to efficiently fabricate reconfigurable electronic and photonic devices and systems using light‐sensitive elements combined with organic DOEs, providing a new direction for the design and fabrication of such devices.

## Experimental Section

##### Silk Solution Preparation

Silk protein solution was prepared by established purification protocols. The sericin of *bombyx moray* cocoons was removed by boiling for 60 min in aqueous 0.02 m Na_2_CO_3_ (Sigma‐Aldrich, USA) and then the Na_2_CO_3_ was washed away with a 3 × 30 rinse process in distilled water. The degummed cocoons were dried in fume hood formore than 12 h and then were dissolved in 9.3 m LiBr (Sigma Aldrich, USA) solution at 60 °C for 4 h. The degummed cocoons solution with residual LrBr was dialyzed for 2 d in distilled water using Slide a Lyzer dialysis cassettes (molecular weight cut off (MWCO) 3500, Pierce, USA) and then centrifuged for 2 × 20 min at 18000 r.p.m. The concentration of silk protein solution was determined by measuring the final dried weight of a specific volume of solution.

##### DOE Design and Optimization

A commercial optical software LightTrans VirtualLab 5 was applied to calculate and simulate diffractive grating on diffractive diffusers that could transfer the incident light beam into arbitrary light patterns. The desired microstructures of diffractive diffusers from bitmap files was transferred to get the diffractive elements presented in geometry data standards II (GDSII) format which was used for the mask to define the pattern of the DOE. In this paper, the wavelength of the incident laser beam was set as 650 nm and the optical setup was chosen as paraxial far field in simulation process. To simplify the fabrication, the phase levels were set as 2 which could be realized by only one phase mask. The pixel size and the pixel size increment were set as 2 µm and 500 nm, respectively. Finally, the calculation and optimization of the microstructure about the DOE were achieved by Iterative Fourier Transform Algorithm.

##### Silicon Master Fabrication

Silicon oxide with 602 nm thickness was grown on a commercial 4 in. silicon wafer. Afterward the standard photolithography was conducted with LC100A photoresist. The microstructures of the DOE were transferred to silicon wafer by the DOE mask. Finally, reactive ion etching was used to etching the silicon oxide.

##### The SRRs Design and Fabrication

The electromagnetic simulations were performed using the finite‐integration time domain solver of the Computer Simulation Technology (CST) Microwave Studio. A single unit of the SRRs is simulated as shown in Figure S8 in the Supporting Information. Perfect magnetic and perfect electric boundary conditions were applied along the x‐ and y‐directions (the gap was along the *y*‐direction). The THz wave was normally incident on the single unit. Then, the structure of the SRRs was fabricated on a commercially purchased “4” high‐resistivity silicon wafer. A standard UV lithography was used to determining the SRRs array and then the Cr/Au layer (10/150 nm) was evaporated on the photoresist. Next, the component mentioned above was performed using a standard liftoff process to form the SRRs.

##### The SRRs with CH_3_NH_3_PbI_3_ Film Fabrication

The SRRs was ultrasonically cleaned and treated with UV‐ozone for 15 min (Figure S15, Supporting Information). The CH_3_NH_3_PbI_3_ film was deposited through a one‐step spin coating process with antisolvent dripping. The PbI_2_ and CH_3_NH_3_I were dissolved in dimethylsulfoxide (99.9%, Sigma‐Aldrich, USA) and N,N‐Dimethylformamide (DMF) with *γ*‐butyrolactone (99.9%, Sigma‐Aldrich, USA) at 1.2 m and stirred until clear. The mixed solution was spinning coating on the SRRs substrate and spun at 1000 and 3000 rpm for 10 and 40 s, respectively. 120 µL of chlorobenzene w/wo conjugated polymer was swiftly dropped onto the substrate at 20 s before the end of the program during the high‐speed spin‐coating step. The SRRs was treated by a heat treatment at 100 °C for 10 min.

##### THz Spectra Measurement

The THz spectra of SRRs with CH_3_NH_3_PbI_3_ film were measured using a commercial THz time‐domain spectroscopy (THz‐TDS) system TAS7500SP (Advantest, Japan) with a scan rate of 8 ms, spectral range from 0.1 to 4 THz. The test cavity was purged by dry air continuously. The SRRs samples were placed by a standard incident angle in which the electric field was perpendicular to the gap of the samples. And the blank high‐resistivity silicon wafer was defined as the reference.

##### Transmission Experimental Setup for DOE Characterization

The silk DOE was illuminated by a commercial laser pointer that was used a laser diode emitting a narrow band coherent laser beam of visible light). The distance between the DOE and the PCB was fixed to be 1.5 m and no collimating/ focusing lens was used.

##### Other Measurement

The electrical signal was supplied by SA‐SG030 signal generator. The measurement of resistors was accomplished by a digit multimeter (Agilent34410A). The output voltage and current were measured using a digital oscilloscope (HMO3002 Series).

## Conflict of Interest

The authors declare no conflict of interest.

## Supporting information

Supporting Information
