# Supplementary material for: Photoinduced Tunable and Reconfigurable Electronic and Photonic Devices Using a Silk‐Based Diffractive Optics Platform
Source: Adv Sci (Weinh). 2020 Jun 4;7(14):2000475. doi: 10.1002/advs.202000475 (PMC7375236; doi:10.1002/advs.202000475)
Supplement: Supplementary file 1 — Supporting Information [file ADVS-7-2000475-s001.pdf]

Copyright WILEY-VCH Verlag GmbH & Co. KGaA, 69469 Weinheim, Germany, 2013.

Supporting Information

Photo-induced Tunable and Reconfigurable Electronic and Photonic Devices Using a Silk-based Diffractive Optics Platform

*Xiaoqing Cai[†], Zhitao Zhou[†], and Tiger H. Tao**

Xiaoqing Cai, Dr. Zhitao Zhou, Prof. Tiger H. Tao
State Key Laboratory of Transducer Technology, Shanghai Institute of Microsystem and Information Technology, Chinese Academy of Sciences, Shanghai 200050, China.
E-mail: tiger@mail.sim.ac.cn

Prof. Tiger H. Tao
Center of Materials Science and Optoelectronics Engineering, University of Chinese Academy of Sciences, Beijing 100049, China

Xiaoqing Cai, Prof. Tiger H. Tao
School of Graduate Study, University of Chinese Academy of Sciences, Beijing 100049, China

Prof. Tiger H. Tao
School of Physical Science and Technology, ShanghaiTech University, Shanghai 200031, China

Prof. Tiger H. Tao
Institute of Brain-Intelligence Technology, Zhangjiang Laboratory, Shanghai 200031, China

Prof. Tiger H. Tao
Shanghai Research Center for Brain Science and Brain-Inspired Intelligence, Shanghai 200031, China

Keywords: Silk, Diffractive optical element, Circuit reconfiguration, Tunable coding metamaterials

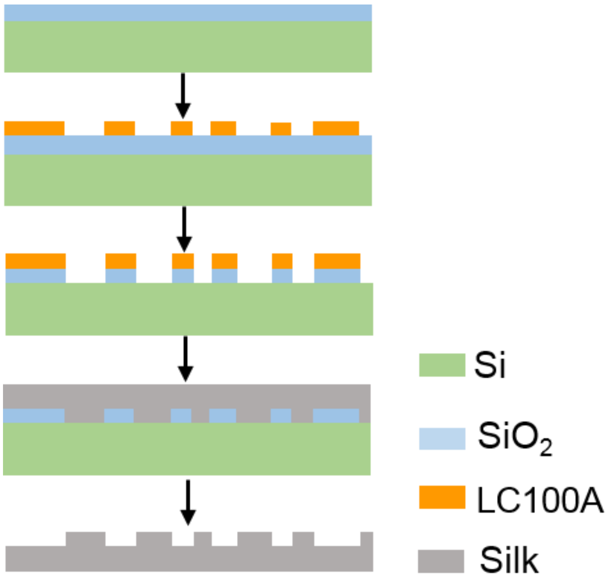

Figure S1. Schematic view of the fabrication of silk-based DOE. The flowchart from top to bottom is growing a layer of SiO2, photoetching, etching the SiO2, casting silk solution on the silicon-based DOE and peeling off the silk-based DOE.

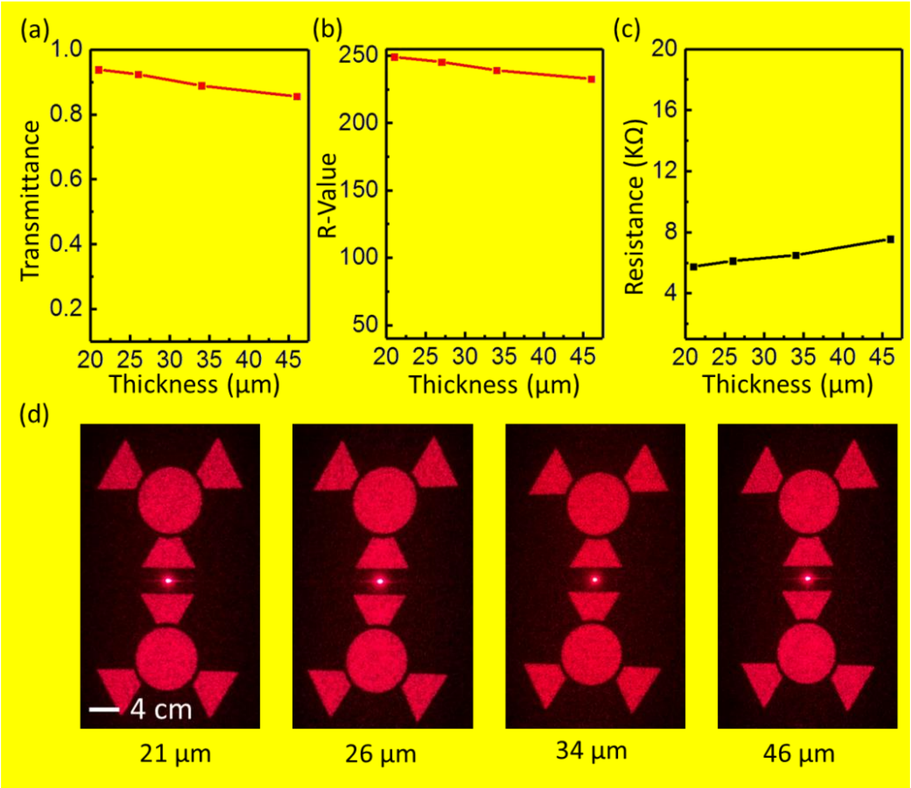

Figure S2. (a) The transmittance of the unpatterned area of silk DOE (working at the wavelength of 650 nm) with different thickness. (b) The R-value of the diffractive patterns. (c) The resistance of photoresistor regulated by the diffractive patterns. (d) The diffractive patterns generated from the silk DOE with different thickness. The thicker thickness of silk-based DOE results in lower transmittance and bigger resistance. However, ultrathin silk film cannot be operated effectively for experiment treatment. Experimental results show that ~46

μm is an ideal thickness, which has satisfactory diffraction property and can be operated effectively.

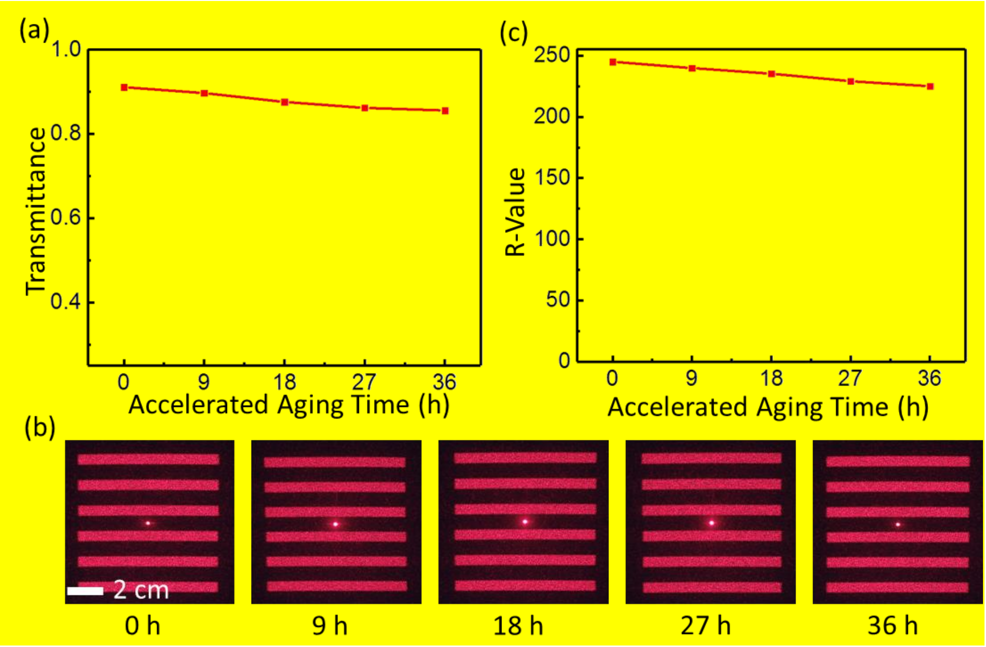

Figure S3. (a) Measured transmittance of the unpatterned area of silk DOE (working at the wavelength of 650 nm) with different accelerated aging time. The (b) diffractive patterns and (c) corresponding R-value of silk DOE obtained at different accelerated aging time points.

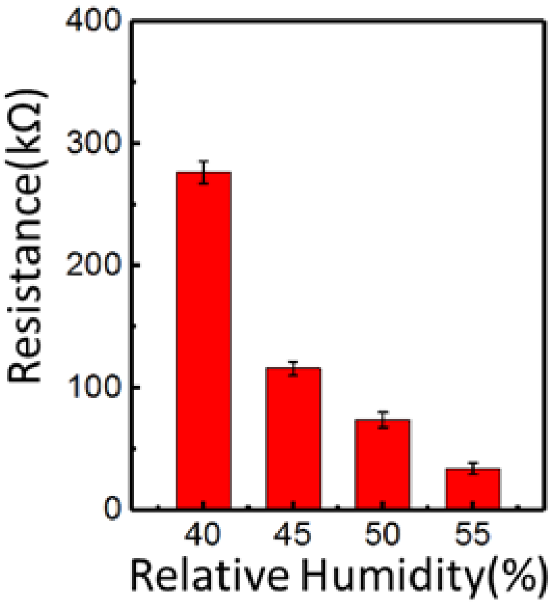

Figure S4. The resistance of R4 measured at relative humidity of 40%, 45%, 50% and 55% at 30℃.

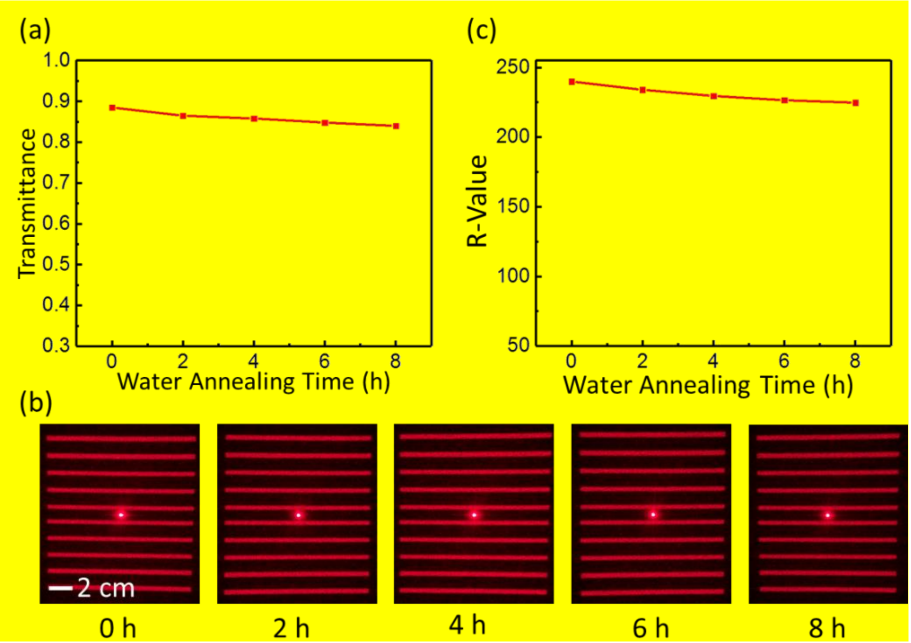

Figure S5. (a) The transmittance of the unpatterned area of silk DOE (working at the wavelength of 650 nm) with different crystallinity levels. (b) The diffractive patterns of silk DOE with different crystallinity levels and (c) the corresponding R-values.

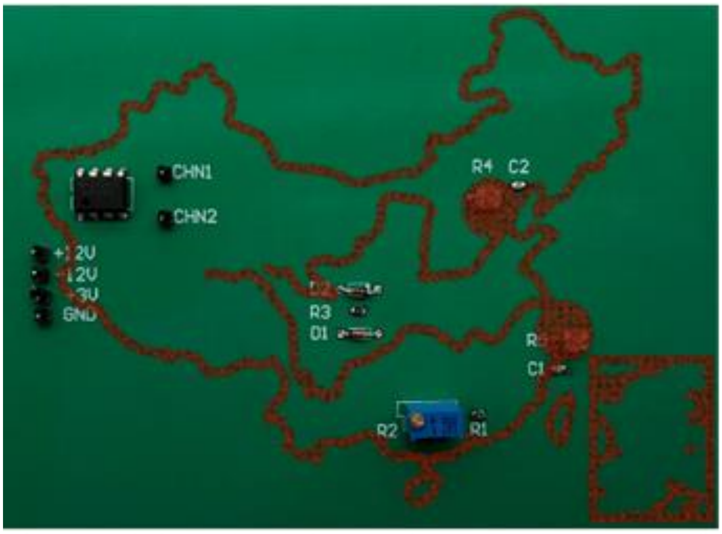

Figure S6. Schematic diagram of diffraction pattern used for regulating oscillation circuit on the PCB. The photoresistors are placed on the locations of “Beijing” and “Shanghai”.

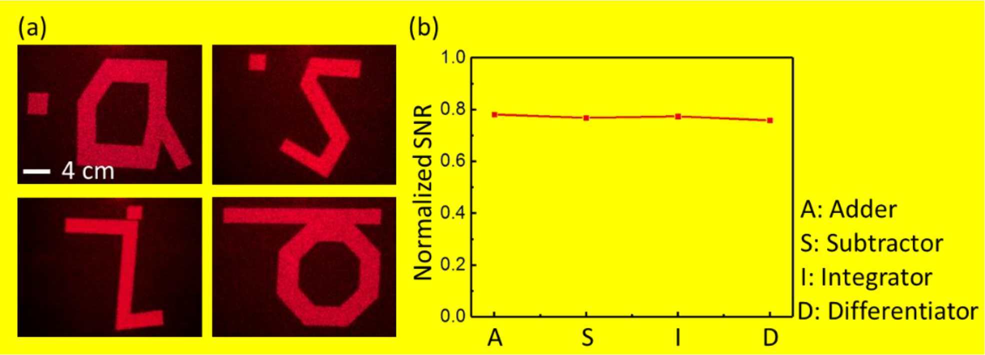

Figure S7. (a) The diffractive patterns and (b) corresponding normalized SNR of the silk DOE working at the wavelength of 650 nm.

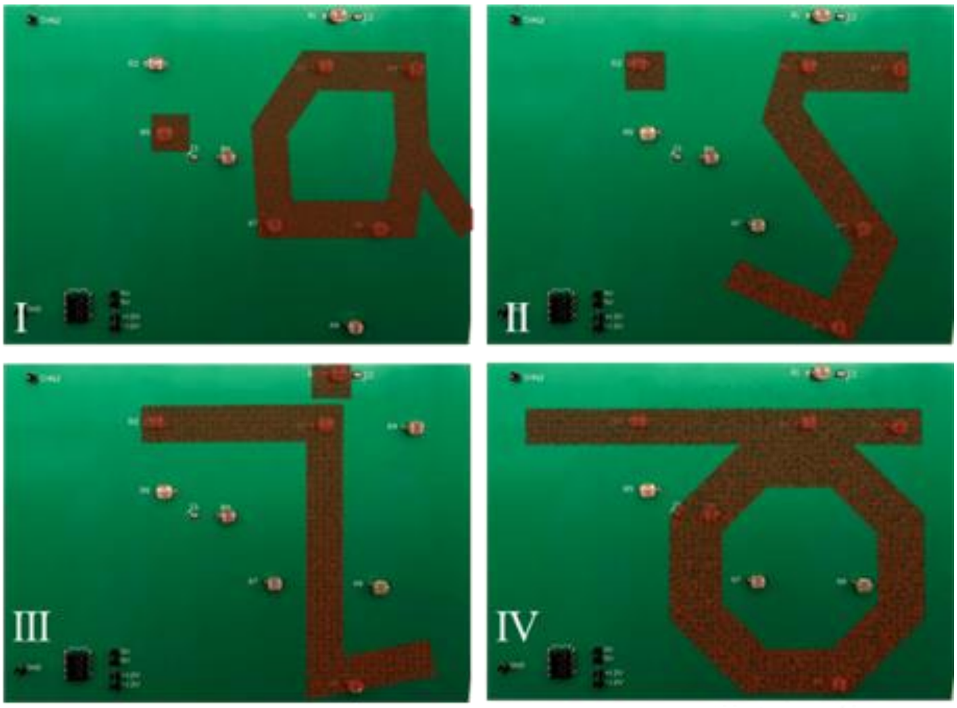

Figure S8. Light diffraction patterns used for reconfiguring adder (I) subtractor (II), integrator (III) and differentiator (IV) on the same PCB, respectively.

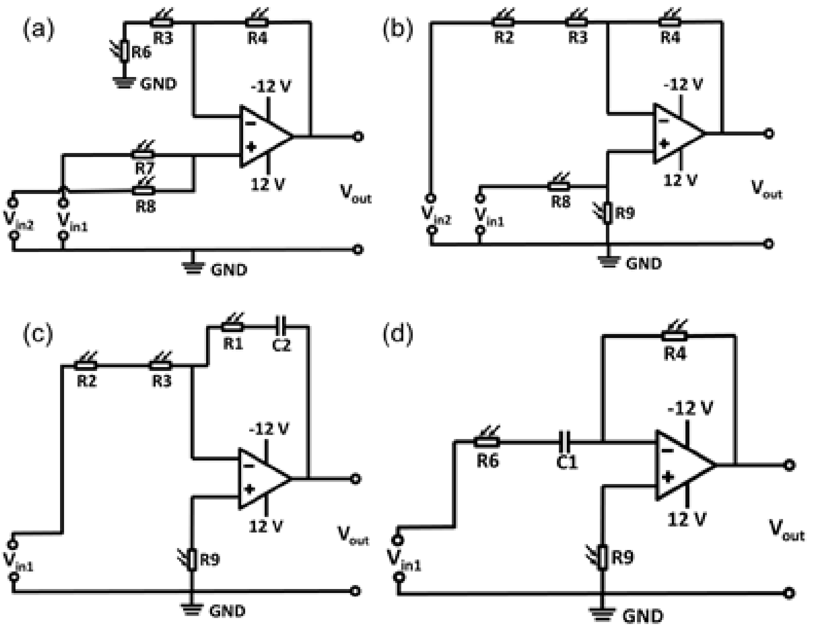

Figure S9. Equivalent circuits of (a) adder, (b) subtractor, (c) integrator and (d) differentiator when illuminating by silk-based DOE, respectively.

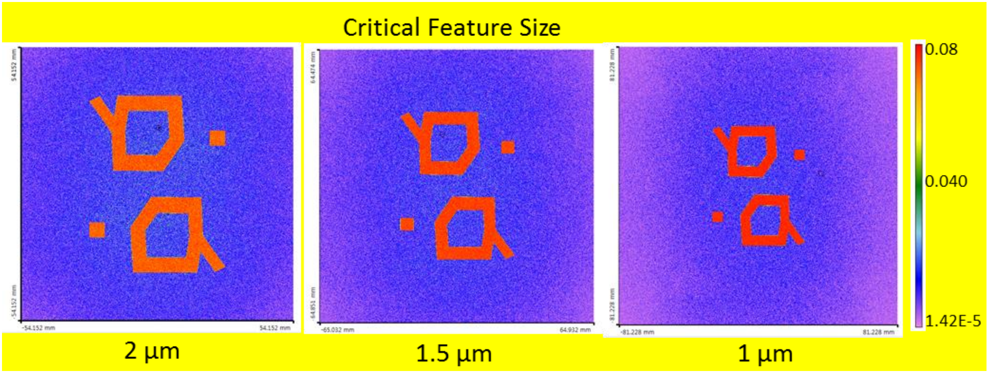

Figure S10. The simulation results of silk DOE with different critical feature sizes (i.e., 2 μm, 1.5 μm and 1 μm).

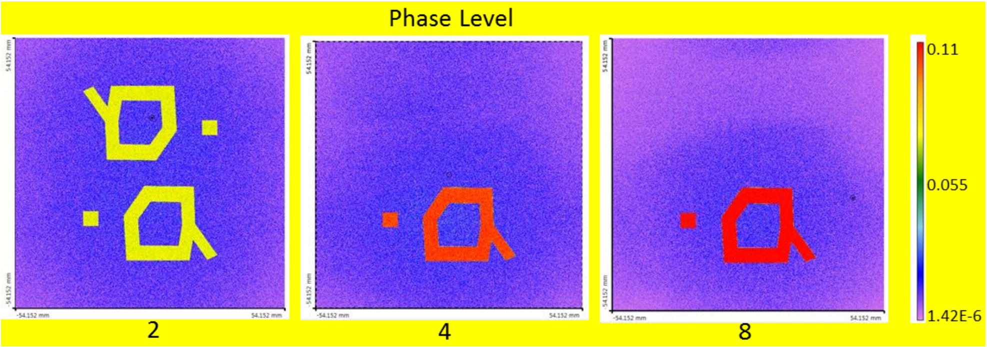

Figure S11. The simulation results of silk DOE with different phase levels (i.e., 2, 4 and 8).

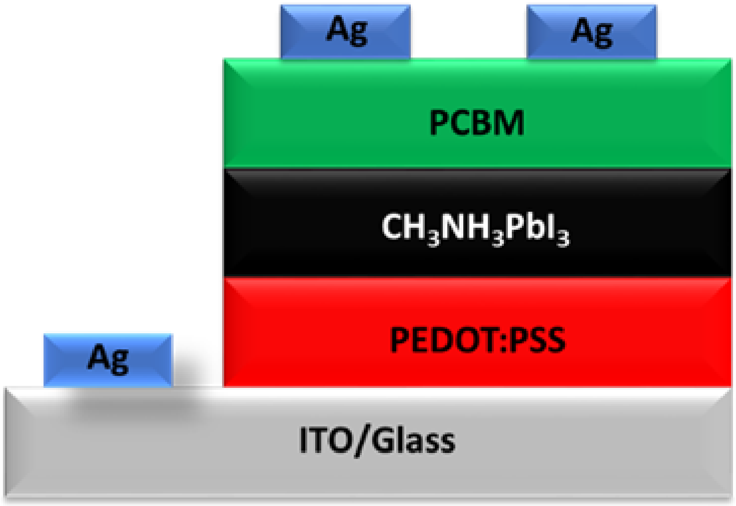

Figure S12. A device structure model used for measuring the properties of perovskite film.

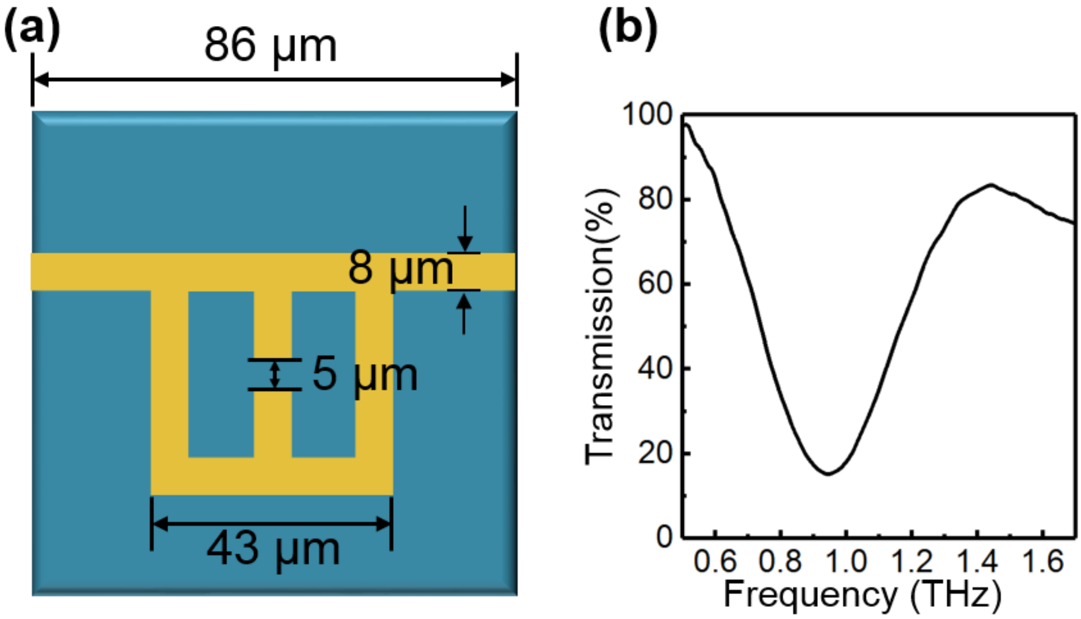

Figure S13. (a) The structure parameters of the SRRs. (b) Measured transmission spectra of SRRs arrays without the perovskite film.

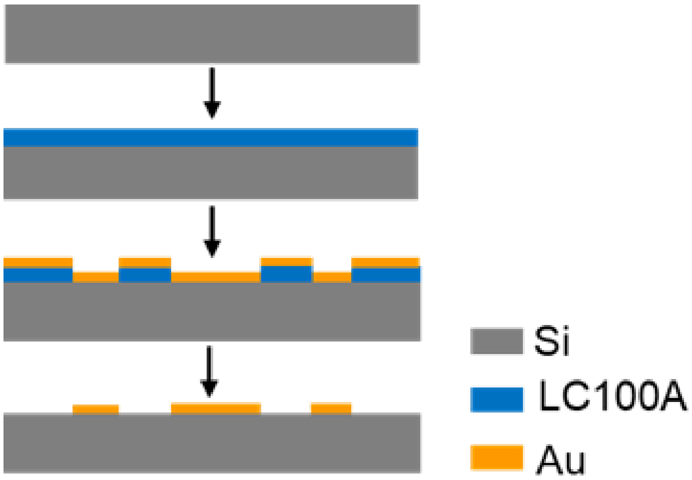

Figure S14. Fabrication process of THz metamaterials on high-resistivity silicon based on metal lift-off technology.

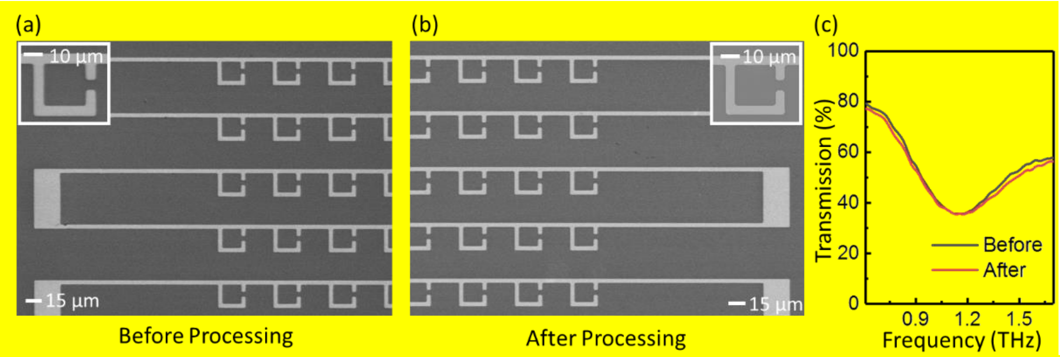

Figure S15. (a, b) The SEM images of the Cr/Au SRRs before and after processing, respectively. (c) Transmission spectra of the Cr/Au SRRs measured before and after processing.
